# Social environment exposure to electronic cigarettes and its association with e-cigarette use among adolescents in Shanghai, China

**DOI:** 10.3389/fpubh.2022.1005323

**Published:** 2022-11-03

**Authors:** Luojia Dai, Weiyi Lu, Juanjuan Wang, Lulu Zhang, Jingfen Zhu

**Affiliations:** School of Public Health, Shanghai Jiao Tong University, Shanghai, China

**Keywords:** e-cigarettes, social environment, exposure, adolescents, tobacco control

## Abstract

**Objective:**

This study investigated adolescents' social-environmental exposure to e-cigarettes in association with e-cigarette use in Shanghai, China. We also explored these differences by gender and school type.

**Methods:**

Sixteen thousand one hundred twenty-three students were included by a stratified random cluster sampling, and the number was weighted according to selection probability. Association between social environment exposure and e-cigarette use was examined by multivariate logistic regressions.

**Results:**

There were 35.07, 63.49, 75.19, 9.44, and 18.99% students exposed to secondhand e-cigarette aerosol (SHA), e-cigarette sales, e-cigarette information, parents' and friends' e-cigarette use. Students exposed to SHA (aOR = 1.73, 95% CI 1.40–2.14), e-cigarette sales from ≥2 sources (aOR = 1.55, 95% CI 1.18–2.03), e-cigarette information exposure from ≥2 sources (aOR = 1.39, 95% CI 1.05–1.83), and having a social e-smoking environment (friends' e-cigarette use: aOR = 2.56, 95% CI 2.07–3.16; parents' e-cigarette use: aOR = 1.54, 95% CI 1.17–2.02) were significantly associated with their intention to use e-cigarettes. More girls were exposed to e-cigarette sales in the malls, e-cigarette information at points of sale and on social media (*P* < 0.01), and exposure to sales from ≥2 sources were associated with girls' intention to use e-cigarettes (aOR = 1.84, 95% CI 1.22–2.78). However, boys were more likely to be exposed to friends' e-cigarette use (*P* < 0.001), and having friends using e-cigarettes was associated with greater intention to use them in boys (aOR = 2.64, 95% CI 1.97–3.55). Less vocational high school students were exposed to parents' e-cigarette use (*P* < 0.001), but they were more likely to use e-cigarettes in the future after being exposed (aOR = 2.27, 95% CI 1.50–3.43). A similar phenomenon was observed between junior high students and their exposure to SHA.

**Conclusions:**

This study reported adolescents' high exposure rates to the social environment of e-cigarettes. Exposure to SHA, e-cigarette sales from ≥2 sources, e-cigarette information from ≥2 sources and having a social e-smoking environment were related to adolescents' intention to use e-cigarettes. Differences in gender and school type were observed. More attention should be paid to girls, and different interventions should be designed for different types of school students. Additionally, comprehensive tobacco control policies are needed.

## Introduction

E-cigarettes have rapidly swept the world over recent years. By featuring various characteristics such as being suitable for use in no-smoking areas, being fashionable, and coming in diverse tastes, they have attracted many adolescents (1). Among current e-cigarette users, adolescents account for ≥20% in countries with a high prevalence of e-cigarettes, such as the United States, the United Kingdom, and Canada (1–3), while the rate of current e-cigarette users in China has also rapidly increased from 1.2% in 2014 to 2.7% in 2019 (4). E-cigarettes might harm adolescents' health, especially when they start using them at such an early stage in life. In the long term, the use of e-cigarettes may lead to a higher risk of cancer, cardiovascular disease, respiratory injury, and osteoporosis (5, 6).

Previous studies have indicated that e-cigarette-related exposure may be the risk factor predisposing adolescents to become current e-cigarette users and try e-cigarettes in the future (7–9). However, more and more adolescents are being exposed to e-cigarettes in their social environment globally *via* secondhand e-cigarette aerosol (SHA), e-cigarette sales, e-cigarette information, and social e-smoking environment. Although some countries, such as Canada, have issued bans prohibiting the advertising of e-cigarettes in mass media, there were still 74% of adolescents who reported being exposed to e-cigarette advertising in 2017. Also, the rate of adolescents was higher (>80%) in the United States and the United Kingdom, where no such ban was implemented (10). Additionally, 29.2 and 27.7% of adolescents in the UK recalled seeing e-cigarettes in supermarkets and retail stores in the past 30 days, respectively, in 2016 (8), while 28.8% of Chinese adolescents reported being exposed to e-cigarette advertising in the past 30 days (11). As for e-cigarette information exposure, previous research in Shanghai, China, also revealed that 73.9% of adolescents knew about e-cigarettes, and the primary sources of information were the internet (42.4%), movies/TV (36.4%), bulletin boards in retail stores or supermarkets (34.9%), advertising flyers (33.9%) (12). In addition, compared to non-e-cigarette users, current users among Chinese teens reported higher rates of friends' smoking (7.2 vs. 0.8%) and parents' smoking (4.9 vs. 1.9%) (13), which is consistent with the situation in the United States (friends' smoking: 32.6 vs. 23.1%; parents' smoking: 38.6 vs. 37.1%) (14). With regard to SHA, 25.6% of US adolescents reported being exposed to it in 2017 (15), vs. 29% of youth from Florida in 2019 (16). However, few studies have addressed SHA exposure among Chinese adolescents.

The sales of e-cigarettes have been increasing for quite some time now (17). For example, the total retail sales of e-cigarettes in the United States increased by 16% from 2015 to 2016 and 47% from 2016 to 2017 (18), thus increasing the likelihood among teens to see related products in vaping stores, convenience stores, supermarkets, and on the internet (19). Though tobacco control compliance is actively promoted in China, the tobacco industry constantly seeks countermeasures. One research reported that 106,485 pieces of online tobacco information were published on 11 different Chinese platforms (20), while another study comparing the web-based e-cigarette information from Google (in English) and Baidu (in Chinese) search engines revealed that more websites on Baidu were owned by manufacturers and were more likely to contain e-cigarette advertising (21). Moreover, in recent years, e-cigarette marketing has shifted from traditional media to social media (8), such as Facebook, Instagram, and YouTube in the United States and Weibo, WeChat, and TikTok in China, all of which are frequently used by adolescents. An earlier study showed that the proportion of adolescents using Facebook, Snapchat, Instagram, and YouTube was 51, 69, 72, and 85%, respectively (22), and 100% of high school students had at least one social account (23). Studies have shown that 30.4% of American junior and senior high school students reported seeing e-cigarette advertisements on social media in the past 30 days (24), while 18.0% reported exposure to e-cigarette information on social media among adolescents in Shanghai, China (12). Social media promote participation, openness, communalization, and connectivity, thus providing a convenient and informal channel for e-cigarette marketing, thus resulting in an unsatisfactory effect of regulations on e-cigarette marketing on social media (10).

Moreover, some specific groups may be especially targeted by e-cigarette marketing. For example, the gender differences in e-cigarette use are much more insignificant than that in traditional smoking (25), and the environmental exposure of girls to e-cigarettes is becoming more and more severe (10, 26). However, since there has been a paucity of related research, in the present study, we described adolescents' exposure to SHA, e-cigarettes information, e-cigarettes sales, and social e-smoking environment, examining the association between social-environmental exposure of e-cigarettes and e-cigarette use among junior, senior, and vocational high school students in Shanghai, China. We also explored the differences in relation to gender and school type.

## Methods

### Research procedure

From June to October 2021, a stratified random cluster sampling was used to select a representative study sample of adolescents aged 13–18 years old in Shanghai. In the first stage, 3 districts in Shanghai were randomly selected, and in the second stage, schools in these districts were selected based on the proportion of junior, senior, and vocational high schools. A total of 21 schools, including 12 junior high schools, 6 senior high schools, and 3 vocational high schools, were randomly selected, and all students in the schools were invited to participate in the study. A total of 16,694 surveys were received, and 16,123 (96.58%) valid questionnaires were included in the analysis. Those with too short answer time and logical contradiction were excluded.

The self-administered questionnaire was adapted from the WHO Global Youth Tobacco Survey. Data were collected by trained investigators. Students were asked to fill out the questionnaires anonymously and independently. All research procedures were approved by the Shanghai Municipal Education Commission and the participating schools. Written informed consent, which was provided before enrollment, was obtained from respondents. The consent included the objectives, procedures, potential risks, and the benefits of the study. This study was approved by the Ethics Committee of the School of Public Health, Shanghai Jiao Tong University (SJUPN-202015; approved on February 20, 2021).

### Measures

#### Socio-demographic factors

The assessed characteristics included gender, school type, boarding situation, school performance, and monthly allowance. School performance was divided into the top 25%, average, and the bottom 25% of the class; monthly allowance was divided into low, medium, and high, where <200 RMB (30 USD) was low, and ≥600 RMB (90 USD) was high.

#### Secondhand e-cigarette aerosol exposure

Exposure to SHA was determined by asking: “During the past 30 days, were you exposed to vapor from an e-cigarette smoked by someone else?” (15), with possible answers: “never,” “sometimes/often.” Respondents who chose a response other than “never” were considered as exposed to secondhand e-cigarette aerosol, and the variable was then recorded as “no” and “yes.”

#### E-cigarette information exposure

Exposure to e-cigarette information was measured by asking: “Have you seen or heard of e-cigarettes from the following sources?.” The sub-items were: “Social media (e.g., QQ, WeChat, Weibo, TikTok etc.),” “Traditional media (e.g., TV/movies/broadcasting, billboards, magazines etc.), “Points of sale (e.g., convenience stores, newsstands, tobacco stores etc.)” (11). Total e-cigarette information exposure was coded as: “no,” “one source,” and “two and more sources.”

#### E-cigarette sales exposure

Exposure to e-cigarette sales was assessed by the following items: “Have you seen e-cigarettes sold in retail stores around your school?,” “Have you seen e-cigarette sold in the malls?,” “Have you seen anyone selling e-cigarettes on social media (e.g., QQ, WeChat, Weibo, TikTok etc.)?” (11). Total e-cigarette sales exposure was coded as: “no,” “one source,” and “two and more sources.”

#### Social e-smoking environment

Adolescents were asked: “Have your parents used e-cigarettes in the past 30 days?” with a “yes “or “no” response; and “How many of your best friends use e-cigarettes?” with responses “none,” “some,” “most,” or “all,” which were then dichotomously re-coded as “no” or “yes”(27).

#### Cigarette and e-cigarette use

Use of cigarettes was measured by asking: “Have you ever tried cigarette smoking?” and “Have you ever smoked in the past 30 days?.” Never smokers were defined as those who reported “I never smoked even just 1 or 2 puffs” to both items. Current smokers were identified as respondents who reported using cigarettes in the past 30 days, and ever smokers were classified as those who reported lifetime using cigarettes while having used it in the past 30 days (28). For e-cigarettes, respondents were asked whether they had tried e-cigarettes and whether they had used e-cigarettes in the past 30 days. “Never e-cigarette users,” “current e-cigarette users,” and “ever e-cigarette users” were identified based on the same approach as smokers above (28). Intention to use e-cigarettes was measured by the following questions: “Would you try e-cigarettes, even just one puff if given the chance?” and “If one of your best friends were to give you one, would you try it?.” Response options were “Definitely yes,” “Probably yes,” “Probably not,” and “Definitely not” (29). Those who reported “Definitely not” on both items were regarded as having no intention to use e-cigarettes, and others were classified as having the intention to use e-cigarettes.

### Statistical analysis

Considering the complexity of survey sample design, a weighing factor was calculated according to the selection probability of districts, the number of schools in each district, and the number of students in each school, and was then adjusted for the non-response. A Chi-square test was used to analyze whether the rates of SHA exposure, e-cigarette sales exposure, e-cigarette information exposure, parents' and friends' e-cigarette use differed by gender and school type. A series of multivariate logistic regressions were conducted to examine whether current e-cigarette use was associated with the social environment of e-cigarette exposure after controlling for gender, school type, boarding situation, school performance, monthly allowance, and traditional smoking status in model 1, while all variates were controlled in model 2. The association between intention to use e-cigarettes and the social environment of e-cigarette exposure was analyzed by multivariate logistic analysis among non-e-cigarette users and was also conducted after stratification by gender and school type in model 2. Adjusted odds ratios (aOR) with 95% confidence intervals (CIs) were calculated. A *p* < 0.05 was considered statistically significant. Data analysis was performed by SPSS 26.0 software (IBM, NY, USA) and R 4.1.2 software.

## Results

### Descriptive statistics

As shown in Table 1, 16,123 respondents were valid, and the weighted number of students in Shanghai in 2021 was 727,524. The overall weighted sample of students from junior high school, senior high school, and vocational high school accounted for 64.66% (95% CI 63.90–65.41%), 22.99% (95% CI 22.35–23.64%), and 12.36% (95% CI 12.00–12.72%), respectively. Their mean age was 14.22 (95% CI 14.18–14.25) years old. There were slightly more male students (53.11%, 95% CI 52.21–54.01%) than female (46.89%, 95% CI 45.99–47.79%), while boarding and local students accounted for 13.86% (95% CI 13.38–14.35%) and 62.92% (95% CI 62.03–63.81%), respectively. A small proportion of students (5.10%, 95% CI 4.73–5.50%) had ever smoked, and a few (1.47%, 95% CI 1.29–1.68%) were current smokers. As for e-cigarette-related behaviors, ever and current e-cigarette users accounted for 3.03% (95% CI 2.75–3.33%) and 0.97% (95% CI 0.83–1.13%), respectively. Moreover, there were more ever, and current male users (4.22%, 95% CI 3.79–4.69%; 1.48%, 95% CI 1.24–1.75%) than female users (1.68%, 95% CI 1.38–2.05%; 0.40%, 95% CI 0.29–0.57%). Meanwhile, the rates of ever, and current e-cigarette users increased in the order of junior (2.31%, 95% CI 1.96–2.73%; 0.46%, 95% CI 0.31–0.67%), senior (3.20%, 95% CI 2.72–3.75%; 1.64%, 95% CI 1.31–2.06%) and high school students (6.47%, 95% CI 5.86–7.14%; 2.42%, 95% CI 2.05–2.85%). Among all students, 6.46% (95% CI 6.06–6.89%) reported having intention to use e-cigarettes.

**Table 1:** Baseline characteristics.

	**Weighted**	**Number**	**Unweighted**
	**Proportion**		
	**%(95% CI)**		
**Age (mean, 95% CI)**
	14.22 (14.18–14.25)	727524	16123
**Gender**
Male	53.11 (52.21–54.01)	382773	8817
Female	46.89 (45.99–47.79)	337940	7306
**School type**
Junior high school	64.66 (63.90–65.41)	466001	5888
Senior high school	22.99 (22.35–23.64)	165666	4566
Vocational high school	12.36 (12.00–12.72)	89046	5669
**Boarding situation**
Yes	13.86 (13.38–14.35)	99893	4190
No	86.14 (85.65–86.62)	620820	11933
**Residence**
Local	62.92 (62.03–63.81)	453482	10610
Non-local	37.08 (36.19–37.97)	267231	5513
**School performance**
Top 25%	34.37 (33.51–35.23)	247689	5402
Average	47.23 (46.33–48.14)	340428	7733
Bottom 25%	18.40( 17.71–19.11)	132597	2988
**Monthly allowance**
Low	60.82 (59.97–61.68)	438373	7749
Medium	27.17 (26.41–27.94)	195828	5407
High	12.00 (11.50–12.53)	86512	2967
**Traditional smoking status**
Never	93.43 (92.99–93.84)	673347	14821
Ever	5.10 (4.73–5.50)	36751	953
Current	1.47 (1.29–1.68)	10615	349
E-cigarette use			
Never	96.00 (95.67–96.31)	691878	15235
Ever	3.03 (2.75–3.33)	21826	649
Current	0.97 (0.83–1.13)	7010	239
**Intention to use e-cigarettes**
No	93.54 (93.11–93.94)	674130	14824
Yes	6.46 (6.06–6.89)	46583	1299

### Social environmental exposure

Table 2 shows students' social-environmental exposure to e-cigarettes and their stratification by gender and school type. Approximately 20% of students (18.99%, 95% CI 18.36–19.65%) reported having friends using e-cigarettes, and nearly 10% (9.44%, 95% CI 8.92–10.00%) had at least one parent using e-cigarettes. In addition, 35.07% (95% CI 34.22–35.94%) reported being exposed to the vapor of someone else's e-cigarette. As for e-cigarette sales exposure, most students (63.49%, 95% CI 62.61–64.36%) were exposed to e-cigarette marketing, where the rates from one source and two and more sources were 42.06% (95% CI 41.17–42.96%) and 21.43% (95% CI 20.71–22.16%), respectively. Additionally, 59.85% (95% CI 58.96–60.73%) of all students were exposed to e-cigarette sales in the malls, 14.15% (95% CI 13.51–14.81%) reported being exposed in the retail stores around school, and 14.10% (95% CI 13.52–14.69%) reported seeing people selling e-cigarettes on their social media. In terms of exposure to e-cigarette-related information, more than 70% of students were exposed, where the rates from one source and from two and more sources were 41.71% (95% CI 40.82–42.61%) and 33.47% (95% CI 32.64–34.32%), respectively. Also, there were 48.36% (95% CI 47.46–49.26%), 37.08% (95% CI 36.21–37.96%), and 37.91% (95% CI 37.06–38.78%) students who were exposed through points of sale, traditional media and social media, respectively.

**Table 2:** Social environment exposure to e-cigarettes among adolescents.

	**Gender**					**School type**				
	**Total**	**Male**	**Female**	**χ2**	***P***	**Junior high school**	**Senior high school**	**Vocational high school**	**χ2**	***P***
	**%(95% CI)**	**%(95% CI)**	**%(95% CI)**			**%(95% CI)**	**%(95% CI)**	**%(95% CI)**		
SHA exposure				2.91	0.144				48.69	<0.001
No	64.93 (64.06–65.78)	64.32 (63.14–65.49)	65.61 (64.34–66.86)			66.49 (65.27–67.69)	63.97 (62.57–65.35)	58.51 (57.22–59.79)		
Yes	35.07 (34.22–35.94)	35.68 (34.51–36.86)	34.39 (33.14–35.66)			33.51 (32.31–34.73)	36.03 (34.65–37.43)	40.21–42.78		
Total e-cigarette sales exposure				19.71	0.001				83.82	<0.001
No	36.51 (35.64–37.39)	37.55 (36.36–38.75)	35.34 (34.07–36.62)			37.19 (35.97–38.44)	34.65 (33.28–36.04)	36.41 (35.17–37.67)		
One source	42.06 (41.17–42.96)	40.44 (39.23–41.67)	43.90 (42.58–45.22)			43.51 (42.25–44.78)	40.17 (38.75–41.60)	38.00 (36.74–39.27)		
Two and more sources	21.43 (20.71–22.16)	22.01 (21.03–23.03)	20.76 (19.72–21.84)			19.29 (18.31–20.32)	25.19 (23.95–26.47)	25.60 (24.48–26.75)		
Sales exposure in the malls				16.83	<0.001				6.91	0.016
No	40.15 (39.27–41.04)	41.64 (40.43–42.86)	38.47 (37.18–39.77)			40.54 (39.29–41.80)	38.37 (36.97–39.79)	41.44 (40.16–42.72)		
Yes	59.85 (58.96–60.73)	58.36 (57.14–59.57)	61.53 (60.23–62.82)			59.46 (58.20–60.71)	61.63 (60.21–63.03)	58.56 (57.28–59.84)		
Sales exposure in retail stores around school				9.71	0.010				55.93	<0.001
No	85.85 (85.19–86.49)	85.05 (84.13–85.93)	86.76 (85.81–87.66)			84.41 (83.46–85.31)	87.71 (86.73–88.63)	89.95 (89.13–90.70)		
Yes	14.15 (13.51–14.81)	14.95 (14.07–15.87)	13.24 (12.34–14.19)			15.59 (14.69–16.54)	12.29 (11.37–13.27)	10.05 (9.30–10.87)		
Sales exposure on social media				4.28	0.057				470.06	<0.001
No	85.90 (85.31–86.48)	85.37 (84.54–86.16)	86.51 (85.64–87.33)			90.25 (89.47–90.98)	78.98 (77.77–80.13)	76.03 (74.90–77.12)		
Yes	14.10 (13.52–14.69)	14.63 (13.84–15.46)	13.49 (12.67–14.36)			9.75 (9.02–10.53)	21.02 (19.87–22.23)	23.97 (22.88–25.10)		
Total e-cigarette information exposure				33.87	<0.001				153.49	<0.001
No	24.81 (24.03–25.61)	26.60 (25.52–27.72)	22.78 (21.67–23.93)			26.36 (25.25–27.50)	21.81 (20.64–23.04)	22.30 (21.23–23.40)		
One source	41.71 (40.82–42.61)	41.22 (40.00–42.45)	42.27 (40.97–43.59)			43.46 (42.20–44.73)	37.45 (36.06–38.86)	40.50 (39.23–41.79)		
Two and more sources	33.47 (32.64–34.32)	32.18 (31.05–33.33)	34.94 (33.70–36.20)			30.18 (29.02–31.37)	40.74 (39.32–42.17)	37.20 (35.95–38.47)		
Information exposure in points of sale				18.71	<0.001				10.71	0.002
No	51.64 (50.74–52.54)	53.24 (52.00–54.47)	49.83 (48.50–51.16)			50.99 (49.71–52.26)	51.69 (50.24–53.13)	54.98 (53.68–56.27)		
Yes	48.36 (47.46–49.26)	46.76 (45.53–48.00)	50.17 (48.84–51.50)			49.01 (47.74–50.29)	48.31 (46.87–49.76)	45.02 (43.73–46.32)		
Information exposure on social media				10.43	0.005				550.73	<0.001
No	62.09 (61.22–62.94)	63.24 (62.07–64.4)	60.77 (59.49–62.04)			68.65 (67.45–69.82)	51.64 (50.19–53.09)	47.17 (45.87–48.47)		
Yes	37.91 (37.06–38.78)	36.76 (35.60–37.93)	39.23 (37.96–40.51)			31.35 (30.18–32.55)	48.36 (46.91–49.81)	52.83 (51.53–54.13)		
Information exposure on traditional media				0.15	0.744				33.64	<0.001
No	62.92 (62.04–63.79)	63.06 (61.86–64.24)	62.77 (61.48–64.04)			64.05 (62.81–65.26)	58.89 (57.46–60.31)	64.53 (63.27–65.76)		
Yes	37.08 (36.21–37.96)	36.94 (35.76–38.14)	37.23 (35.96–38.52)			35.95 (34.74–37.19)	41.11 (39.69–42.54)	35.47 (34.24–36.73)		
Parents' e-cigarette use				3.69	0.109				31.16	<0.001
No	90.56 (90.00–91.08)	90.97 (90.21–91.67)	90.08 (89.25–90.86)			89.72 (88.92–90.47)	91.31 (90.45–92.09)	93.51 (92.84–94.12)		
Yes	9.44 (8.92–10.00)	9.03 (8.33–9.79)	9.92 (9.14–10.75)			10.28 (9.53–11.08)	8.69 (7.91–9.55)	6.49 (5.88–7.16)		
Friends' e-cigarette use				48.29	<0.001				1316.51	<0.001
No	81.01 (80.35–81.64)	78.99 (78.07–79.88)	83.29 (82.36–84.18)			89.22 (88.40–89.98)	67.87 (66.50–69.21)	62.48 (61.21–63.73)		
Yes	18.99 (18.36–19.65)	21.01 (20.12–21.93)	16.71 (15.82–17.64)			10.78 (10.02–11.60)	32.13 (30.79–33.50)	37.52 (36.27–38.79)		

When stratified by sex, results showed that female students were more likely to be exposed to sales in the malls (χ^2^ = 16.828, *P* < 0.001), e-cigarette information at points of sale (χ^2^ = 18.714, *P* < 0.001), and on social media (χ^2^ = 10.428, *P* < 0.01). Also, males were more likely to be exposed to sales in retail stores around the school (χ^2^ = 9.712, *P* < 0.05) and friends' e-cigarette use (χ^2^ = 48.290, *P* < 0.001).

All kinds of social-environmental exposure to e-cigarettes differed by school type (*P* < 0.05). Additionally, the rates of exposure to SHA (χ^2^ = 48.69, *P* < 0.001), e-cigarette sales exposure through social media (χ^2^ = 470.06, *P* < 0.001), information exposure through social media (χ^2^ = 550.73, *P* < 0.001) and friends' e-cigarette use (χ^2^ = 1316.51, *P* < 0.001) increased in the order of junior, senior, and vocational high school, while the rates of exposure to e-cigarette sales in retail stores around the school (χ^2^ = 55.93, *P* < 0.001), information in points of sale (χ^2^ = 10.71, *P* < 0.01), and parents' e-cigarette use (χ^2^ = 31.16, *P* < 0.001) decreased in the same order.

### Associations between social-environmental exposure and e-cigarette use

As shown in Table 3, after adjusting for socio-demographic factors and traditional smoking status in model 1, results showed that students who were exposed to SHA, parents' and friends' e-cigarette use, and e-cigarette sales were more likely to currently use and to express intention to use them in the future (*P* < 0.01). Moreover, greater sales exposure was related to higher odds of adolescents' intention to use. However, after adjusting for all variates in model 2, only students exposed to e-cigarette sales from two and more sources were significantly associated with current use and intention to use it (aOR_current_ = 5.68, 95% CI 2.91–11.09, aOR _intention_ = 1.55, 95% CI 1.18–2.03). Students who were exposed to SHA were significantly associated with current e-cigarette use (aOR = 2.18, 95% CI 1.28–3.69) and greater intention of using (aOR = 1.73, 95% CI 1.40–2.14). Moreover, friends' e-cigarette use was mostly associated with these e-cigarette-related behaviors (aOR _current_ = 5.44, 95% CI 3.06–9.66, aOR _intention_ = 2.56, 95% CI 2.07–3.16). Positive associations were also found between parent's e-cigarette use and students' current use and intention to use e-cigarettes (aOR_current_ = 7.28, 95% CI 4.75–11.14, aOR _intention_ = 1.54, 95% CI 1.17–2.02). With respect to e-cigarette information exposure, a positive association was only found between exposure from two and more sources and students' intention to use (aOR = 1.39, 95% CI 1.05–1.83).

**Table 3:** Association between social-environmental e-cigarette exposure and adolescents' e- cigarette use and intention.

	**Current e-cigarette use** ^**a**^				**E-cigarette use intention** ^**b**^			
	**Model 1^c^**	***P***	**Model 2^d^**	***P***	**Model 1^c^**	***P***	**Model 2^d^**	***P***
	**aOR(95% CI)**		**aOR(95% CI)**		**aOR(95% CI)**		**aOR(95% CI)**	
**SHA exposure**
No	Ref = 1		Ref = 1		Ref = 1		Ref = 1	
Yes	6.35 (3.95–10.20)	<0.001	2.18 (1.28–3.69)	0.004	2.68 (2.23–3.21)	<0.001	1.73 (1.40–2.14)	<0.001
**Total e-cigarette sales exposure**
No	Ref = 1		Ref = 1		Ref = 1		Ref = 1	
One source	2.87 (1.52–5.43)	0.001	1.57 (0.76–3.22)	0.220	1.62 (1.28–2.04)	<0.001	1.15 (0.89–1.47)	0.284
Two and more sources	11.73 (6.68–20.61)	<0.001	5.68 (2.91–11.09)	<0.001	2.73 (2.15–3.46)	<0.001	1.55 (1.18–2.03)	0.002
**Total e-cigarette information exposure**
No	Ref = 1		Ref = 1		Ref = 1		Ref = 1	
One source	1.22 (0.76–1.96)	0.403	0.97 (0.54–1.74)	0.918	1.29 (0.99–1.68)	0.062	1.16 (0.88–1.53)	0.290
Two and more sources	1.21 (0.75–1.94)	0.439	0.60 (0.33–1.07)	0.083	1.98 (1.53–2.55)	<0.001	1.39 (1.05–1.83)	0.020
**Parents' e-cigarette use**
No	Ref = 1		Ref = 1		Ref = 1		Ref = 1	
Yes	9.91 (6.72–14.61)	<0.001	7.28 (4.75–11.14)	<0.001	1.99 (1.53–2.59)	<0.001	1.54 (1.17–2.02)	0.002
**Friends' e-cigarette use**
No	Ref = 1		Ref = 1		Ref = 1		Ref = 1	
Yes	11.26 (6.40–19.81)	<0.001	5.44 (3.06–9.66)	<0.001	3.55 (2.92–4.32)	<0.001	2.56 (2.07–3.16)	<0.001

^a^Among all participants (*N* =727,524).

^b^Among non-e-cigarette users (*N* = 691,878).

^c^Model adjusted for gender, school type, boarding situation, residence, monthly allowance, school performance and traditional smoking status.

^d^Model adjusted for gender, school type, boarding situation, residence, monthly allowance, school performance, traditional smoking status, SHA exposure, e-cigarette sales exposure, e-cigarette information exposure, parents' e-cigarette use and friends' e-cigarette use.

### Stratification of association between social-environmental exposure and e-cigarette use intention by gender and school type

**Figure 1:**
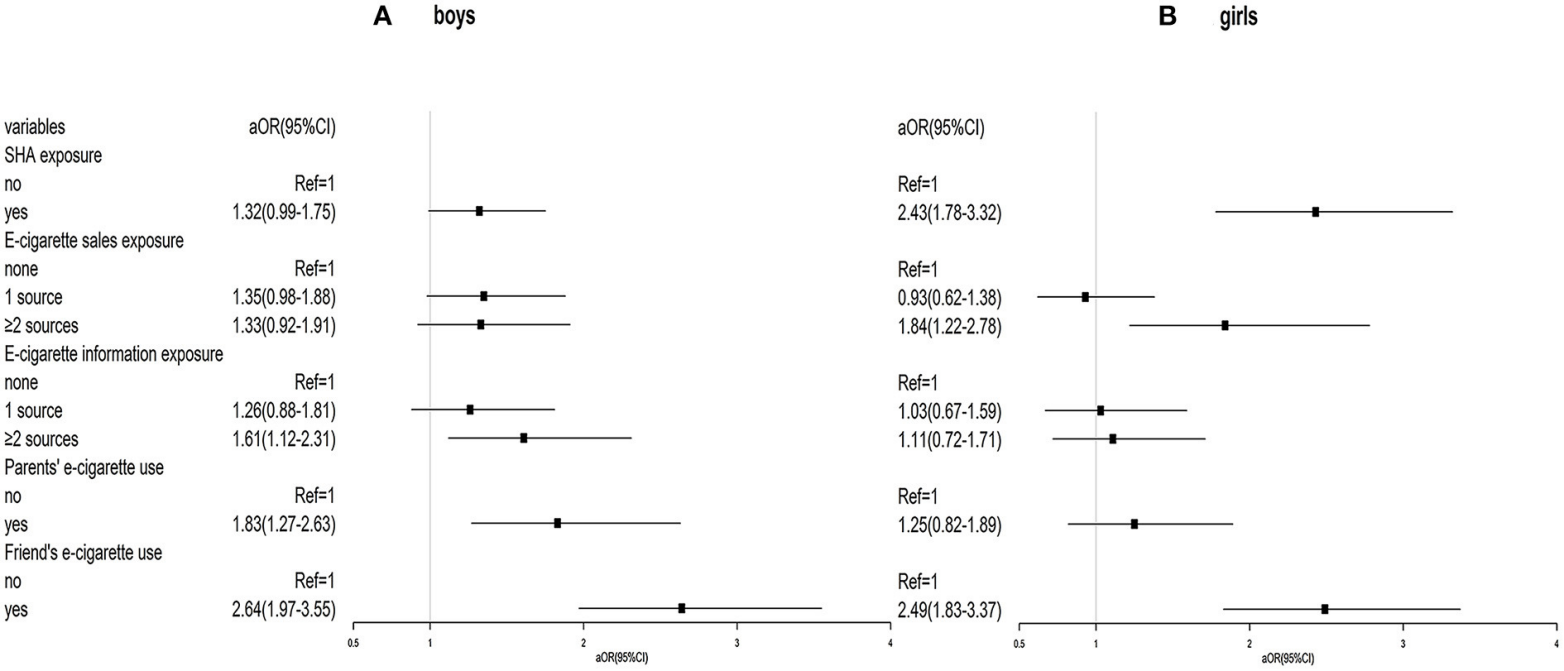
**(A,B)** Stratification of association between social-environmental exposure and e-cigarette use by gender.

When stratified by gender, female non-e-cigarette users were more likely to use e-cigarettes in the future when exposed to SHA (aOR = 2.43, 95% CI 1.78–3.32) and e-cigarette sales from two and more sources (aOR = 1.84, 95% CI 1.22–2.78). However, having friends using e-cigarettes (aOR = 2.64, 95% CI 1.97–3.55) was associated with greater intention to use e-cigarettes in boys compared to girls (aOR = 2.49, 95% CI 1.83–3.37). Also, only boys were significantly associated with intention to use e-cigarettes when exposed to e-cigarette information from two and more sources (aOR = 1.83, 95% CI 1.27–2.63) and parents' e-cigarette use (aOR = 2.64, 95% CI 1.97–3.55) (Figure 1).

**Figure 2:**
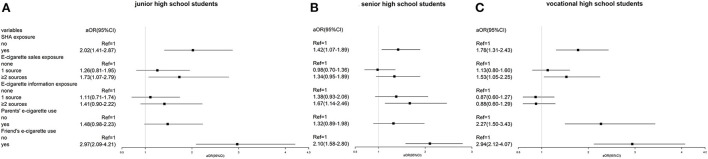
**(A–C)** Stratification of association between social-environmental exposure and e-cigarette use by school type.

As shown in Figure 2, junior high school students were more likely to use e-cigarettes when exposed to SHA (aOR = 2.02, 95% CI 1.41–2.87), e-cigarette sales from two and more sources (aOR = 1.73, 95% CI 1.07–2.79) and friends' e-cigarette use (aOR = 2.97, 95% CI 2.09–4.21). A positive association was only found between e-cigarette information exposure from two and more sources and intention to use among senior high school students (aOR = 1.67, 95% CI 1.14–2.46). Parents' e-cigarette use was only significantly associated with intention to use among vocational high school students (aOR = 2.27, 95% CI 1.50–3.43). Moreover, having friends using e-cigarettes was associated with the greatest intention to use among all students.

## Discussion

This study reported social-environmental exposure and its association with e-cigarette use in adolescents from China. It was found that the social-environmental exposure to e-cigarettes among adolescents in Shanghai was not optimistic, with the rate of e-cigarette sales exposure (63.49%, 95% CI 62.61–64.36%) and the rate of information exposure (75.19%, 95% CI 74.39–75.97%) being especially high. Exposure to SHA, e-cigarette sales, and social e-smoking environment was positively associated with adolescents' current e-cigarette use. Moreover, exposure to SHA, e-cigarette sales from ≥2 sources, e-cigarette information from ≥2 sources and having a social e-smoking environment were significantly related to teenagers' intention to use e-cigarettes, while these associations differed by gender and school type.

In recent years, the Chinese government has highlighted the importance of protecting minors from e-cigarettes, and setting up relevant laws and regulations. According to “*Circular on further protection of minors from e-cigarettes*” issued by the State Administration for Market Regulation and the State Tobacco Monopoly Administration, all e-cigarette sales websites were to be shut down and e-cigarette advertisements posted on the internet withdrawn (30). However, the present study found that the rates of e-cigarette information and sales exposure *via* social media were 37.91% (95% CI 37.06–38.78%) and 14.10% (95% CI 13.52–14.69%), respectively. One study that analyzed the data from the Texas Adolescent Tobacco and Marketing Surveillance System revealed that 52.5% of students were exposed to e-cigarette-related social media in the past month (31). What's unique about e-cigarette information on social media was that they were mostly posted by individual users in various forms, such as push articles, videos, posts forwarded by friends, advertisements etc. (31), and were mostly viewed by their followers. E-cigarette users' positive comments on products on social media, the sharing of interesting e-smoking tricks, and the display of e-cigarettes as fashionable items may lower adolescents' perception of e-cigarettes as something harmful (11) and may arouse their curiosity (29), thus increasing the chances of future e-cigarette use (32). Moreover, it was found that viewing peers' posts on social media were associated with susceptibility to use e-cigarettes (33). On the other hand, the unofficial ways of selling e-cigarettes online may lead to more adverse outcomes, such as purchasing e-cigarettes of unknown origin, as well as issues with e-cigarette product control, which may cause traumatic injury due to self-exploded batteries or self-combusted assembled devices (34).

Accordingly, due to the ban on official online sales, China is now at the stage where offline e-cigarette stores are seizing the market and rapidly expanding, which leads to a high exposure rate of store sales and information. However, previous research indicated that recalled exposure to point-of-sale cigarette displays and advertisements was associated with more frequent cravings to smoke (35). It is necessary to strengthen the implementation of the sign “*Minors are not allowed to buy e-cigarettes*” posted in prominent locations in stores, implement measures such as controlling age and identity cards, and strictly enforce the law, i.e., once minors are found to have purchased e-cigarettes, no matter what the reason, businesses should be punished. In addition, store location and density may also have a role in the prevention of minors from using e-cigarettes (36), as previous studies reported that frequent convenience store access and e-cigarette marketing were risk factors for e-cigarette susceptibility and initiation (37). Restricting e-cigarette shops from prominent positions in major shopping malls and reducing the number of e-cigarette vendors should also be considered. The placement of e-cigarettes at stores has been rarely discussed in China. However, a few states in America have issued legislation prohibiting the self-service of e-cigarettes (38). Regulatory efforts to control the placement of e-cigarettes, thus limiting youth exposure, such as requiring products to be placed in clerk-assisted locations, should be examined (39). As for exposure to e-cigarette sales around the school, the newly revised “*Law of the People's Republic of China on the Protection of Minors”* has established that there should be no e-cigarette retail stores around schools (40). This measure may have a certain effect; however, it does not specify the specific distance and store density, e.g., “Shops selling e-cigarettes are not allowed within 100 meters of the primary and secondary schools” in Beijing's tobacco control regulations can be a reference for consideration (41). The establishment of tobacco control regulations nationwide and the improvement of minors' protection laws are also needed. Moreover, our results showed that the risk of having the intention to use e-cigarettes was relatively higher in students exposed to e-cigarette sales and information from two and more sources, suggesting that more comprehensive e-cigarette management policies are needed to minimize youth exposure to e-cigarette sales and information.

In the present study, we also identified adolescents' social e-smoking environment, showing that the rates of adolescents having parents' and friends' using e-cigarettes were 9.44% (95% CI 8.92–10.00%) and 18.99% (95% CI 18.36–19.65%), respectively. However, previous studies found that 42.4% (42) and 19.2% (43) of American youth reported having friends and parents who were using e-cigarettes. Consistent with earlier research, parents' and friends' e-smoking was significantly associated with adolescents' current e-cigarette use and may elevate the risk of intention to use among never users (43). It is possible that parents' and friends' use and positive attitude may be interpreted as social approval and permissive norms on e-cigarettes, leading to their use without fear of repercussions (42). As for SHA, the exposure rate in this study was 35.07% (95% CI 34.22–35.94%), which was higher than in American youth in 2019 (16). Additionally, SHA, which can lead to death by asthma, lower respiratory infections, and ischemic heart disease (44), is not only harmful to the overall health but may also elevate adolescents' susceptibility to use e-cigarettes (16). Given that regulations on e-cigarettes are relatively loose in China, potential increase for e-cigarette use among Chinese adolescents should be considered.

Similar with what was like among adults (45), the rates of ever and current e-cigarette users among boys were higher than those among girls in our study. Moreover, they were more likely to have friends using e-cigarettes and have intention to use it after being exposed. Therefore, tobacco control education and peer intervention for male students are of great importance. However, unlike traditional cigarette enterprises, females are the main target of e-cigarette marketing. The current e-cigarette use rate (0.4%) among the girls in this study was much higher compared to females aged 15–24 (0.1%) (45), while that rate among boys (1.5%) who were current e-cigarette users was lower than in males aged 15–24 (2.7%) (45), which suggests that preventing e-cigarette use among teenage girls is of crucial importance. Our results revealed that the rates of e-cigarette sales in the malls and information exposure were significantly higher among girls than boys, which is consistent with the findings in other countries. For example, Canadian females were more likely to be exposed to e-cigarette tricks on social media (46) and a significant higher prevalence of exposure to any e-cigarette advertisement was found among American girls than boys (47). Moreover, girls expressed greater intention to use e-cigarettes when exposed to sales from two and more sources. Thus, specific efforts should be made to lower their exposure to e-cigarette sales and information. For example, in addition to banning stores from selling e-cigarettes to female minors, it should also be considered not allowing e-cigarette stores to be set up on the girls' clothing floor of the shopping malls. E-cigarette advertising should not be allowed to use colorful and fashionable images, to set girls-targeted themes (e.g., “girls' night”), and to feature slim, sexy and attractive female models. Meanwhile, though no difference was found in exposure to SHA between genders, girls exposed to SHA were much more likely to use e-cigarettes. Previous research also found that among non-e-cigarette users, girls were more likely to be susceptible (46). Thus, there might be more female e-cigarette users in the future without proper intervention, which also calls for more attention on lowering girls' social environment exposure to e-cigarettes to prevent their use.

Social-environmental exposure to e-cigarettes also differed among adolescents of different school types. Students from vocational high schools were more likely to be exposed to friends' e-cigarette use, SHA, e-cigarette sales, and information exposure through social media. This may be related to the lower academic pressure and less strict school management. Special attention should be paid to the establishment of smoke-free schools in vocational high schools. Additionally, parents' and friends' e-cigarette use were the most relevant to vocational high students' intention to use e-cigarettes. Offline exposure, such as sales and information exposure in retail stores around the school, was found to be higher among junior high school students, which might be because younger adolescents are less likely to be allowed to use electronic devices, thus becoming the target of offline marketing. Moreover, students from junior high school were more likely to use e-cigarettes in the future when exposed to SHA, e-cigarette sales from two and more sources, and friends' e-cigarette use, which might be because younger teenagers are more likely to use to be influenced by their surroundings. E-cigarette exposure interventions should have different priorities for different types of school students. For example, intervention on vocational high school students should focus on their social e-smoking environment, and measures on lowering junior high school students' offline e-cigarette exposure should be promoted.

There are several limitations in the present study. First, data were self-reported and are subject to recall bias; thus, the rates of adolescents' social environment exposure to e-cigarettes may be under- or over-estimated. Second, since this was a cross-sectional study, a causal relationship could not be inferred. However, the odds ratios presented in this study remained significant after controlling for all variates, which strongly predicts adolescents' intention to use e-cigarettes when being exposed to such a social environment. Longitudinal data are critically needed. Third, as e-cigarette information exposure was assessed by a single item, unmeasured confounders or mediators might be neglected (e.g., level of social media use, pro or con of e-cigarettes conveyed by the information). Finally, the data from this study are representative of social environment exposure to e-cigarettes in Chinese urban cities, but not the overall situation in China. Nevertheless, Shanghai is the most economically developed mega-city in China, where adolescents are more likely to be exposed to new and fashionable products; therefore, prevention and control in Shanghai can provide a reference for other cities and regions.

## Conclusions

Overall, this study found that social-environmental exposure to e-cigarettes was high among adolescents in Shanghai, China. Exposure to SHA, e-cigarette sales from ≥2 sources, e-cigarette information from ≥2 sources and having a social e-smoking environment were related to their intention to use e-cigarettes. Moreover, more attention should be paid to girls, and relevant intervention measures should be tailored based on different priorities for different types of school students. Since e-cigarette is unsafe to adolescents and may lead to traditional smoking, comprehensive tobacco control policies, including efforts to prevent youth exposure to SHA, e-cigarette sales, information, and social e-smoking environment, should be made to prevent e-cigarette use among youth.

## Data availability statement

The raw data supporting the conclusions of this article will be made available by the authors, without undue reservation.

## Ethics statement

The studies involving human participants were reviewed and approved by the Ethics Committee of the School of Public Health, Shanghai Jiao Tong University. Written informed consent to participate in this study was provided by the participants' legal guardian/next of kin.

## Author contributions

JZ and WL conceived and designed the study. LD and JZ analyzed the data. LD, JW, and WL drafted the manuscript. WL, LZ, and JZ collected the data. All authors contributed to revise the paper and approved the final manuscript.

## Funding

This study was funded by National Social Science Foundation of China (Grant No. 20BSH133).

## Conflict of interest

The authors declare that the research was conducted in the absence of any commercial or financial relationships that could be construed as a potential conflict of interest.

## Publisher's note

All claims expressed in this article are solely those of the authors and do not necessarily represent those of their affiliated organizations, or those of the publisher, the editors and the reviewers. Any product that may be evaluated in this article, or claim that may be made by its manufacturer, is not guaranteed or endorsed by the publisher.
